# Estimating axon conduction velocity *in vivo* from microstructural MRI

**DOI:** 10.1016/j.neuroimage.2019.116186

**Published:** 2019-12

**Authors:** Mark Drakesmith, Robbert Harms, Suryanarayana Umesh Rudrapatna, Greg D. Parker, C. John Evans, Derek K. Jones

**Affiliations:** a Cardiff University Brain Research Imaging Centre, Cardiff University, Cardiff, United Kingdom; b Neuroscience and Mental Health Research Institute, Cardiff University, Cardiff, United Kingdom; c Department of Cognitive Neuroscience, Faculty of Psychology and Neuroscience, Maastricht University, the Netherlands; d Phillips Inovation Campus, Bangalore, India; e Experimental MRI Centre (EMRIC), School of Biosciences, Cardiff University, Cardiff, United Kingdom; f Mary McKillop Institute for Health Research, Faculty of Health Sciences, Australian Catholic University, Melbourne, Victoria, 3065, Australia

**Keywords:** conduction velocity, Axon diameter, Myelin, G-ratio, Diffusion MRI, Relaxometry MRI, Tissue micro-structure, Biophysical modelling, White matter, Axons, Action potentials, Optic nerve, Corpus callosum

## Abstract

The conduction velocity (CV) of action potentials along axons is a key neurophysiological property central to neural communication. The ability to estimate CV in humans *in vivo* from non-invasive MRI methods would therefore represent a significant advance in neuroscience. However, there are two major challenges that this paper aims to address: (1) Much of the complexity of the neurophysiology of action potentials cannot be captured with currently available MRI techniques. Therefore, we seek to establish the variability in CV that *can* be captured when predicting CV purely from parameters that have been reported to be estimatable from MRI: inner axon diameter (AD) and g-ratio. (2) errors inherent in existing MRI-based biophysical models of tissue will propagate through to estimates of CV, the extent to which is currently unknown. Issue (1) is investigated by performing a sensitivity analysis on a comprehensive model of axon electrophysiology and determining the relative sensitivity to various morphological and electrical parameters. The investigations suggest that 85% of the variance in CV is accounted for by variation in AD and g-ratio. The observed dependency of CV on AD and g-ratio is well characterised by the previously reported model by Rushton. Issue (2) is investigated through simulation of diffusion and relaxometry MRI data for a range of axon morphologies, applying models of restricted diffusion and relaxation processes to derive estimates of axon volume fraction (AVF), AD and g-ratio and estimating CV from the derived parameters. The results show that errors in the AVF have the biggest detrimental impact on estimates of CV, particularly for sparse fibre populations (AVF<0.3). For our equipment set-up and acquisition protocol, CV estimates are most accurate (below 5% error) where AVF is above 0.3, g-ratio is between 0.6 and 0.85 and AD is high (above 4μm). CV estimates are robust to errors in g-ratio estimation but are highly sensitive to errors in AD estimation, particularly where ADs are small. We additionally show CV estimates in human corpus callosum in a small number of subjects. In conclusion, we demonstrate accurate CV estimates are possible in regions of the brain where AD is sufficiently large. Problems with estimating ADs for smaller axons presents a problem for estimating CV across the whole CNS and should be the focus of further study.

## Highlights

• 85% of the variance in CV is accounted for by axon diameter and g-ratio, which are potentially accessible from *in vivo* MRI.
• CV estimates from MRI are robust to errors in myelin and axonal volume estimates, but sensitive to errors in axon diameter.
• CV estimates are feasible for large axons but limitations of *in vivo* imaging of small axons poses a significant challenge.

## 1 Introduction

The conduction velocity (CV) of action potentials along axons is a key neurophysiological property upon which neural communication depends. While *in vivo* CV measurements in peripheral nerves are comparatively trivial, it is currently not possible to obtain *in vivo* estimates of CV in the central nervous system (CNS). The ability to make such estimates, however, would yield a great deal of insight into how the brain encodes and integrates information and how such mechanisms are optimised in the human brain (Pumphrey and Young, 1938; Omi and Shinomoto, 2008; Carr and Konishi, 1988; Sugihara et al., 1993; Budd et al., 2010; Schüz and Preil, 1996; Ford et al., 2015; Innocenti, 2017). Furthermore, being able to image CV in CNS axons *in vivo* would allow us to identify individual differences in CV, and examine how and why CV is altered in healthy development, ageing and disease states.

Previously, simple relationships between axon morphology and CV have been derived from early electrophysiological and theoretical literature (Gasser and Grundfest, 1939; Hursh, 1939; Huxley and Stämpeli, 1949; Rushton, 1951; Smith and Koles, 1970; Waxman and Bennett, 1972; Moore et al., 1978; Tolhurst and Lewis, 1992) (see Waxman (1980) for a review). In particular, Rushton (1951) derived a very simple relationship: $v\propto Dg\sqrt{-\text{ln}\left(g\right)}\propto d\sqrt{-\text{ln}\left(g\right)}$, where *d* is the inner axon diameter, *D* is the outer fibre (axon plus myelin sheath) diameter and *g* is the ratio between the two, g=d/D. An alternative model derived by Waxman and Bennett (1972) models CV as a simple linear function of outer fibre diameter: v∝D∝d/g. The constant of proportionality of this relationship usually falls in the range of 5.5–6.0 $\text{m}\;{\text{s}}^{-1}/\text{μm}$ and is often used as a simple way of predicting CV from fibre diameter (Caminiti et al., 2013; Tomasi et al., 2012; Innocenti et al., 2014).

Recent developments in MRI acquisition technology and modelling claim to provide non-invasive estimates of microstructural attributes relevant to CV, including axon diameter (AD) (Assaf et al., 2008; Alexander et al., 2010), axonal volume fractions (AVF) (Assaf and Basser, 2005; Zhang et al., 2012), myelin volume fractions (MVF) (Deoni et al., 2008; West et al., 2016; Duval et al., 2017; Campbell et al., 2018) and g-ratios (Stikov et al., 2015; Dean et al., 2016). It is tempting, therefore, to speculate that one might use this information to obtain individual-specific estimates of CV *in vivo*. The literature is currently sparse regarding attempts to do this. Horowitz et al. (2015), showed a correlation between MRI-based estimates of AD and inter-hemispheric transfer delay measured with electroencephalography, implying MRI-derived estimates of AD correlate with CV. A more recent study is that of Berman et al. (2019). Here, g-ratios were estimated in human corpus callosum from macromolecular tissue volume (MTV) estimates of myelin and diffusion based-estimates of axonal volume fraction. AD was not derived from the same subjects but extrapolated from existing histology data (Aboitiz et al., 1992). This approach showed slightly slower CVs in older subjects compared to slower subjects. However, the authors conclude that individual-specific estimates of AD would be essential for modelling individual-specific CV.

These studies assume simple relationships between axonal microstructural parameters and CV. However, beyond these parameters, CV depends, to a greater or lesser extent, on a number of parameters that are not currently accessible *in vivo*, and yet contribute considerable variability across fibre populations and across individuals. These include the distance between the nodes of Ranvier, inter-nodal spacing, and electrical properties of the axonal and myelin membranes. We address these issues through simulation and then present some CV estimates in human corpus callosum obtained from *in vivo* MRI data.

## 2 Sensitivity of CV to axonal parameters

This section addresses the first issue: How sensitive is CV to axonal parameters and are the simplified models of CV sufficient to capture variance in CV when many other relevant parameters are inaccessible to *in vivo* MRI?

The physiological mechanisms of an axon's action potential propagation have a complex dependency on many parameters that cannot be quantified *in vivo*. In particular, microstructural properties of the nodes of Ranvier, including their length and diameter, contribute to the surface area on which permeable ion channels can reside, impacting on the electrical properties of the axon. Moreover, the inter-nodal distance is important in determining how many instances of depolarisation are required for an action potential to traverse a unit length of axon. Given these various factors, it is important to establish whether it is feasible to obtain accurate estimates of CV from a simplified model using only parameters that have previously been reported as being quantifiable using MRI.

A sensitivity analysis on parameters affecting CV has previously been performed (Moore et al., 1978). However, this utilised a simple one-at-a-time (OAAT) analysis (where each parameter is varied one at a time) which does not consider *combinations* of parameters, and how interactions between parameter changes affect CV. Moreover, a number of important properties that affect the excitation of the axonal membrane, such as the peri-axonal space, were omitted in that previous analysis. Here, we perform a more comprehensive analysis. We perform extensive simulations of axon physiology using the model of Richardson et al. (2000) and perform sensitivity analysis to determine the sensitivity of CV across a wide region of the parameter space, and to quantify the variance in CV accounted for by each parameter.

### 2.1 Method

#### 2.1.1 Core electrophysiological simulations

The ‘Model C’ axon model of Richardson et al. (2000), as implemented by Arancibia-Cárcamo et al. (2017) (code obtained from https://github.com/AttwellLab/MyelinatedAxonModel) was used to analyse the sensitivity of CV to variance in each of the 14 parameters listed in the upper part of Table 1. Model parameters derived from optic nerve (Arancibia-Cárcamo et al., 2017; Brinkmann et al., 2008) were used as a proxy for CNS axons. Some parameters were assumed to be well-constrained across individuals and fibre populations and thus not tested (fixed parameters listed in Table 1). Others, such as the number of myelin wraps and myelin thickness, are dependent on g-ratio, AD and myelin periodicity, and so were not directly manipulated. The simulated axon was comprised of 50 laminated internodal regions. All parameters were kept constant across all nodes and internodes along the length of the axon. Some internode parameters, such as periaxonal width, were varied at the paranode to accommodate unique morphological characteristics in these regions (see Mierzwa et al. (2010) for further details).

**Table 1:** Baseline and range values for each parameter tested for parameters of interest and values for fixed parameters of the Richardson model. All baseline values were those used in Arancibia-Cárcamo et al. (2017). values for ranges were obtained from [1] Arancibia-Cárcamo et al. (2017) or [2] Brinkmann et al. (2008) otherwise 20% of the baseline value was used.

Varied parameters			
Parameter	Units	Baseline value ($\phi_{i}$) [1]	± Limits ($\left|\text{Δ}\phi_{i}\right|$)
Axon diameter	μm	0.82	0.31 [1]
Node diameter	μm	0.73	0.19 [2]
Node length	μm	1.02	0.15 [1]
Internode length	μm	139.26	31.53 [1]
Peri-axonal width	nm	15	3
Myelin periodicity	nm	15.6	3.12
g-ratio	–	0.78	0.057 [2]
Internode leakage conductance	$\text{mS}{\text{cm}}^{-2}$	0.1	0.02
Intra-axonal resistivity	Ωm	0.7	0.14
Peri-axonal resistivity	Ωm	0.7	0.14
Myelin conductance	$\text{mS}{\text{cm}}^{-2}$	1	0.2
Fast Na ​+ ​conductance	$\text{mS}{\text{cm}}^{-2}$	30	6
Persistent Na ​+ ​conductance	$\text{mS}{\text{cm}}^{-2}$	0.05	0.01
Slow K+ conductance	$\text{mS}{\text{cm}}^{-2}$	0.8	0.16
**Parameters dependent on varied parameters**			
Parameter	Units	Baseline value	
Myelin width	μm	0.101	
Myelin periodicity	nm	15.6	
# of myelin wraps	–	7	
Node diameter	μm	0.73	
Internode diameter	μm	1.05	
Paranode diameter	μm	1.02	
Paranode length	μm	2.11	
Periaxonal width at internode	nm	15	
Effective periaxonal width at paranode	nm	0.0077	
**Fixed parameters**			
Temperature	Co	37	
Number of nodes	–	50	
Stimulus amplitude (baseline condition)	nA	2.73	
Stimulus duration	ms	10	
Axon capacitance	$\text{μF}{\text{cm}}^{-2}$	0.9	
Node capacitance	$\text{μF}{\text{cm}}^{-2}$	0.9	
Myelin membrane capacitance	$\text{μF}{\text{cm}}^{-2}$	0.9	
Node Resting potential	mV	−82	
Node Reversal potential	mV	−83.4	
Na ​+ ​reversal potential	mV	50	
K+ reversal potential	mV	−84

**Table 2:** Goodness of fit statistics for candidate simplified models to data generated from the Richardson model.

Model	SSE	$R^{2}$	Adj. $R^{2}$	RMSE	*k*	AIC	BIC
Rushton model	$2.25\times 10^{3}$	0.993	0.993	4.35	1	1.99	4.78
Linear outer diameter model	$1.07\times 10^{4}$	0.965	0.965	9.48	1	1.96	4.75
Polynomial expansion (full)	29.8	0.9999	0.9998	0.52	10	20.00	47.87
Polynomial expansion (cross-terms only)	$1.43\times 10^{3}$	0.995	0.995	3.49	3	6.00	14.36

**Table 3:** Acquisition parameters used for simulations of diffusion and relaxometry MRI data and for *in vivo* data acquisition.

Parameter	Value
**Diffusion acquisitions**	
Flip angle	$90^{o}$
Slice thickness	2 mm
Field of View	220×220 mm
Matrix size	110×110
Voxel size	2×2×2 mm
CHARMED	
*b*	[500,1200,2400,4000,6000] $\text{s}{\text{mm}}^{-2}$
# directions	[30,60,60,60,60]
*δ*	7 ms
Δ	23.3 ms
Echo time	48 ms
Repetition time	2600 ms
AxCaliber	
$G_{max}$	290 $\text{mT}\;{\text{m}}^{-1}$
*b*	optimised to achieve 100% and 50% of $G_{max}$
# directions	[30,60]
*δ*	7 ms
Δ	[17.3,30,42,55] ms
Echo time	80 ms
Repetition time	3900 ms
**Relaxometry acquisitions**	
Slice thickness	1.72 mm
Field of View	220×220 mm
Matrix size	128×128
Voxel size	1.72×1.72×1.72
SPGR	
Flip angles	[3,4,5,6,7.5,9,12,15,18] ^*o*^
Echo time	1.9 ms
Repetition time	4.2 ms
IR-SPGR	
Flip angle	5°
Echo time	1.9 ms
Repetition time	4.2 ms
Inversion Time	450 ms
SSFP	
Flip angles	[10,10,15,15,20,20,30,30,40,40,50,50,60,60] ^*o*^
Phase cycle angles	[0,180,90,270,0,180,90,270,0,180,90,270,0,180,90,270] ^*o*^
Echo time	2.27 ms
Repetition time	4.54 ms
Slice thickness	1.72 mm

With the exception of geometric interdependencies, all parameters were assumed to be independent, with two exceptions (1) the relationship between the node diameter and inter-node axon diameter. and (2) the relationship between axon diameter and the inter-nodal length (INL) Hursh (1939); Vizoso and Young (1948); Murray and Blakemore (1980); Friede and Bischhausen (1980); Smith et al. (1982); Ibrahim et al. (1995). Since these relationships are well established in the literature, reproducing these relationships in the simulations will ensure greater ecological validity.

The nodal axon diameter was modelled as a linear function of the (inter-nodal) axon diameter:

(1) $$\phi_{ND}=\alpha_{ND}d+\beta_{ND}$$

The INL was modelled as a log relationship with the outer fibre diameter.

(2) $$\phi_{INL}=\alpha_{INL}\;\log\left(\frac{d}{g}+\gamma_{INL}\right)+\beta_{INL}$$

The coefficients $\alpha_{i}$, $\beta_{i}$ $\gamma_{i}$ were optimised to fit a simulated joint distribution of ADs and g-ratios (n=1000, mean and s.d. matched those listed in Table 1) such that the mean and s.d. of the node diameter and INL also match those listed in Table 1. The coefficients obtained for node diameter were $\alpha_{ND}=0.63,\beta_{ND}=0.21$ and for INL were $\alpha_{INL}=159.16,\beta_{INL}=153.74,\gamma_{INL}=-0.227$. In the simulations, each model axon was subjected to a current stimulation applied for 10 ​s to the first node. The amount of current was calibrated such that it produced a peak membrane depolarisation of +50 ​mV in the first node (in the baseline condition, this results in a stimulus amplitude of 2.73 ​nA). The resultant CV was then obtained over a 10-node interval between the 30th and 40th node, except in cases where the CV was too slow for action potentials to reach the 40th node in the simulation duration, in which case the recording interval was moved to earlier segments so that CVs could be obtained. To establish that action potentials propagated consistently along the length of the axon, simulations were checked to ensure membrane potential peaks of at least −40 mV were achieved on a minimum of 10 consecutive nodes.

#### 2.1.2 Sensitivity analysis

Sensitivity was assessed by sampling the corners of a 14-dimensional hypercube in the parameter space, i.e., for every possible combination of positive and negatives changes in each parameter, the CV was simulated and the difference computed, along that dimension of the hypercube. The dimensions of the hypercube were set to 1 s.d. around the baseline condition (with baseline being the same conditions used for the simulations in Arancibia-Cárcamo et al. (2017), given in Table 1), where s.d. was determined from experimental observations in optic nerve (Arancibia-Cárcamo et al., 2017; Brinkmann et al., 2008), or 20% where no such data were available. An exhaustive analysis of $2^{14}=16,384$ comparisons were made. All simulations ran generated action potentials that propagated along the length of the axon.

An OAAT sensitivity analysis was performed for each parameter at 10 equally-spaced intervals within a 20% range around the baseline condition (see Appendix B). This shows that relative changes in CV are approximately linear with change in parameter so we can assume that sampling only the corners of the hypercube is sufficient to capture the variability in CV within this region of the parameter space. The change in CV, $\text{Δ}v\left(\text{Φ}\right)=v{\left(\text{Φ}\right)}^{+}-v{\left(\text{Φ}\right)}^{-}$, for a given set of parameters Φ, due to a change in each individual parameter $\text{Δ}\phi_{i}=\phi_{i}^{+}-\phi_{i}^{-}$, relative to the CV of the baseline condition v(Φ') was calculated. This resulted in $2^{14-1}=8,162$ relative changes. The proportional variance was computed by taking the sum of these changes and normalising to the total variance. The corresponding sensitivity was calculated by normalising the relative change in CV to the relative change in the parameter.

(3) S(ϕi)=Δv(Φ)/v(Φ')Δϕi/ϕ'i

#### 2.1.3 Testing simplified models of CV

We aimed to derive a simple model to predict CV (and associated variance) from the two parameters that have previously been reported as accessible from *in vivo* MRI: g-ratio, and AD. We tested the model across a grid comprising 10 approximately equally-spaced AD values (0.5–12.5 μm) and 12 equally-spaced values of *g* (0.4–0.95). For each grid-point, we repeated the hypercube sensitivity analysis by running the Model C (Richardson et al., 2000) simulation across all possible combinations of the remaining non-MRI accessible parameters, to generate a distribution of CVs for each point on the grid. This resulted in $10\times 12\times 2^{12}=491,520$ model runs. The mean and s.d. of CV at each point was calculated. We then fitted simplified models based on the Rushton formula (Rushton, 1951) and the linear relationship with outer diameter (Waxman and Bennett, 1972) to the mean CV values. We also explored some more complex polynomial models that could potentially provide better fits to the data. In all cases, metrics of the model fit performance and parsimony, including Akaike (1973) and Bayesian (Schwarz and Schwarz, 1978) information criteria (AIC and BIC) were computed, using the likelihood values computed from $\ln\hat{L}=\left(1-R^{2}\right)/2$.

### 2.2 Results

**Fig. 1:**
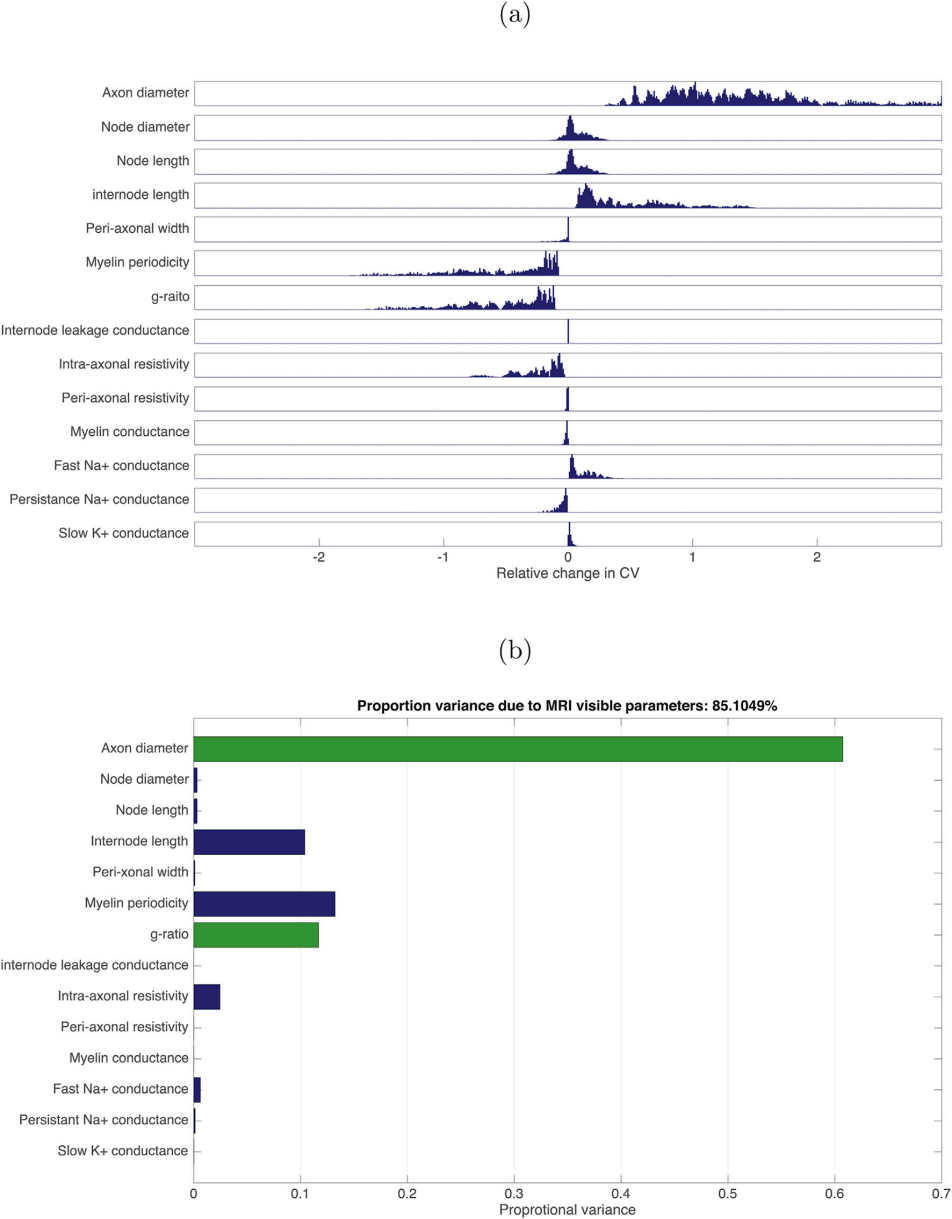
(a) Distributions of relative change in CV for a stepwise change in each parameter (parameter step size determined by limits indicated in Table 1) across all points tested in the parameter space (8,162). (b) The total variance for each parameter step change as proportion of variance across all simulations. MRI-visible parameters indicated by green bars.

**Fig. 2:**
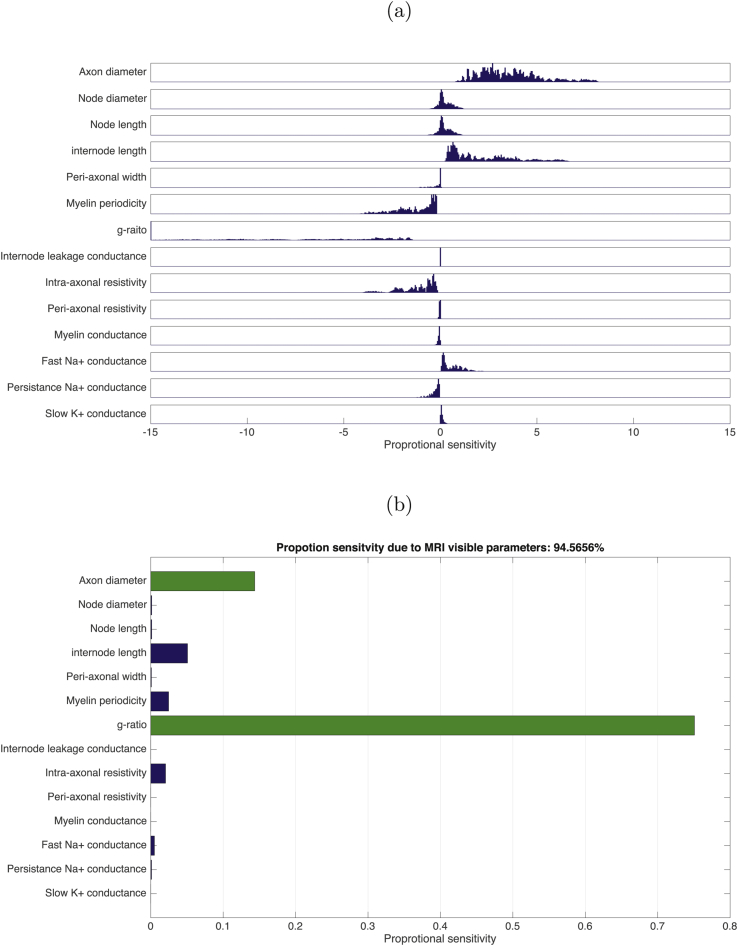
(a) Distributions of relative sensitivities of CV to unit change in each parameter across all points in the parameter space (8,162). (b) The sum-squared relative sensitivity for each parameter step change as proportion of the total sum-squared sensitivity across all simulations. MRI-visible parameters indicated by green bars.

The conduction velocity obtained in the baseline condition was 2.95${\text{ms}}^{-1}$, in agreement with the original simulations in Arancibia-Cárcamo et al. (2017) (see also Appendix A for further validation). Action potential propagation was successful in all simulations. The distribution of relative changes in CV, due to change in each parameter, is shown in Fig. 1(a), while Fig. 1(b) shows the total variances in CV due to change in each parameter relative to the total variance. The majority of the variance is explained by AD, followed by myelin periodicity, g-ratio and internodal length. A key finding of this analysis is that combined together, AD and g-ratio explain 85.1% of the model variance in CV. The distribution of relative sensitivities of CV to unit changes in each parameter are shown in Fig. 2(a) while Fig. 2(b) shows the sum-squared sensitivity for each parameter, proportional to the sum-squared sensitivity across all parameters. CV is most sensitive to a unit change in g-ratio by a considerable margin. AD has the second highest sensitivity. Combined together, AD and g-ratio account for 94.6% of the total sensitivity of CV.

**Fig. 3:**
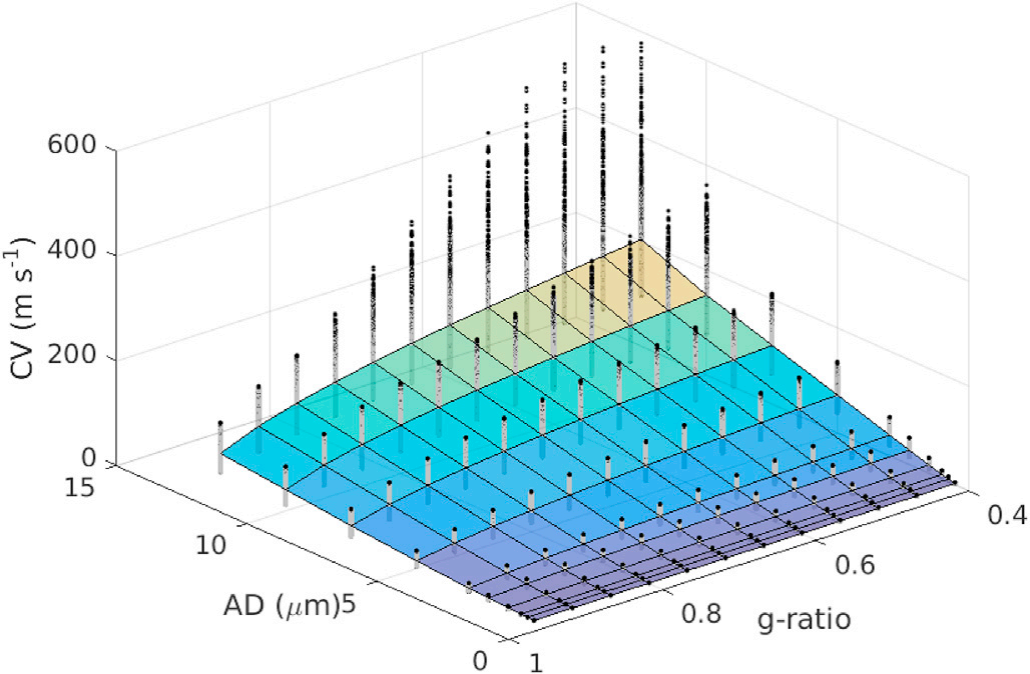
Distribution of CV estimates for each fixed values of AD and g-ratio, across the remaining 12 parameter ranges. Surface plot indicates the mean value. Black dots show the distribution of CV estimates at each point.

**Fig. 4:**
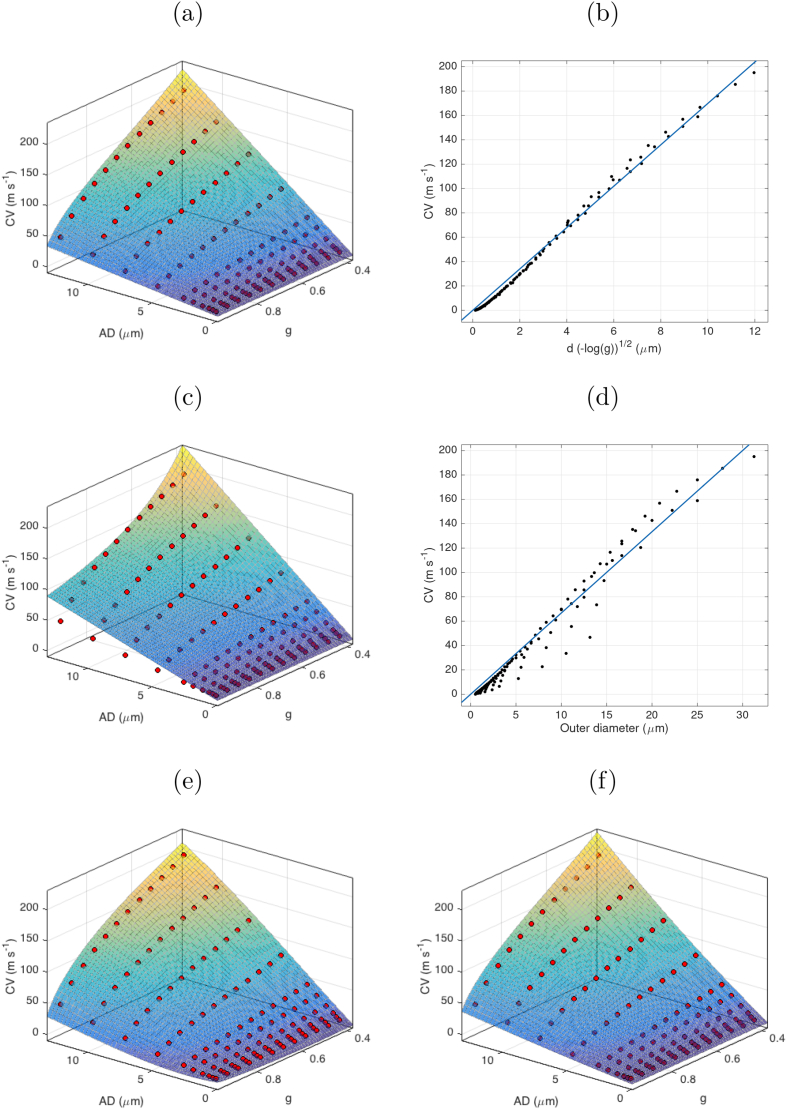
Plots of simplified models fitted to simulated data points across AD and g-ratio values (mean for each AD-g pair indicated by red circles). (a) Rushton model as fitted across values of AD and *g*; (b) Rushton model as a linear fit to $d\sqrt{-log\left(g\right)}$; (c) outer diameter model as fitted across values of *d* and *g*; (d) outer diameter model as a linear fit to outer diameter (d/g); (e) Full 3rd order polynomial expansion in *d* and $\sqrt{-log\left(g\right)}$; (f) the same polynomial expansion only considering the cross-terms.

The distribution of CVs across AD and g-ratio are shown in Fig. 3. The mapping of CV to AD appears approximately linear, while the mapping to *g* follows an inverse log square root function. This is similar to the form given in the Rushton formula. (Rushton, 1951).

(4) $$v=pd\sqrt{-\text{log}\left(g\right)}$$

where *p* is some constant of proportionality, which we estimated in our data to be p=16.99 (confidence bounds: [16.8,17.2]). The 2D fitting to the original Rushton model yielded a good fit (sum squared error (SSE) = $2.247\times 10^{3}$, $R^{2}=0.993$), but the fit was poor where AD is large and g-ratio is small (Fig. 4).

We also tested whether CV could be predicted from a linear function of outer diameter (Waxman and Bennett, 1972). This is simpler to calculate since it uses only one parameter but implicitly assumes a constant *g*-ratio.

(5) $$v=p\frac{d}{g}$$

where *p* was estimated from our data to be p=6.67 (confidence bounds: [6.50,6.85]). Note, the goodness of fit was slightly poorer for this model compared to the Rushton model (SSE = $1.07\times 10^{4}$, $R^{2}=0.964$). The AIC and BIC were comparable to the Rushton model.

**Fig. 5:**
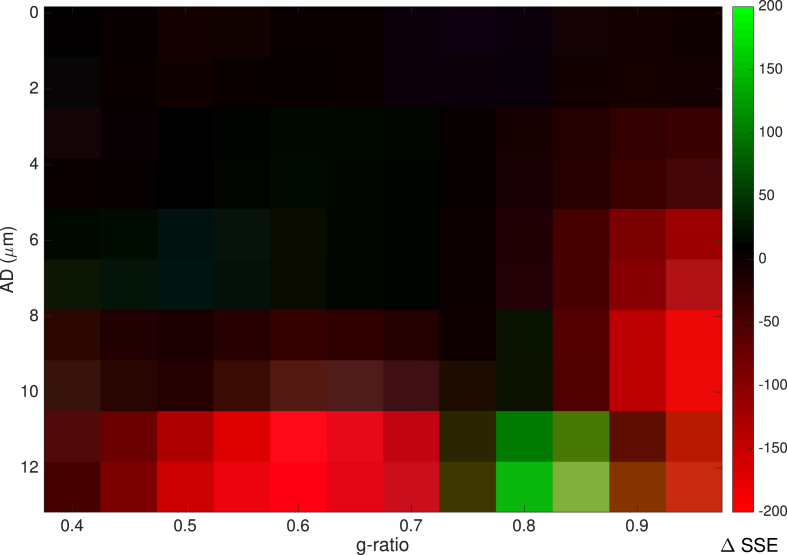
Difference in SSE between the Rushton and linear outer-diameter models. Positive values (green) show higher SSE for The Rushton model, negative values (red) show higher SSE for the outer diameter model.

Further comparison was made between the two models by computing the SSE for each AD-g pair and plotting the difference in SSE (Fig. 5). This shows that where AD is high (above 8μm), there is a better fit (lower SSE) for the Rushton model where g-ratio lies between 0.5 and 0.75. The outer diameter model shows better fit where g-ratio lies between 0.75 and 0.95. Below ADs of 8μm, there is little difference between the two models.

Two more complex models were tested to compare with the Rushton and linear outer diameter models. A 3rd order 2D polynomial expression in *d* and $\sqrt{-\text{log}\left(g\right)}$ yielded a better fit (SSE = 29.8$R^{2}=0.9999$) but required fitting of 10 coefficients. A good fit was also achieved when considering only cross-terms in the same polynomial, (SSE = $1.43\times 10^{3}$$R^{2}=0.995$) which only requires 3 coefficients.

(6) $$v=p_{11}d\sqrt{-\text{log}\left(g\right)}+p_{21}d^{2}\sqrt{-\text{log}\left(g\right)}-p_{12}d\;\log\left(g\right)$$

However, the AIC and BIC are lowest for the Rushton model. Therefore, this remains the preferred model for predicting CV (Fig. 4). The s.d. of the modelled CVs scaled linearly with the mean CV (coefficient = 0.350, SSE = 945.9, $R^{2}=0.977$).

## 3 Estimating CV from MRI-derived parameters

The second part of this study focuses on the second issue highlighted in the introduction: Is it possible to obtain accurate CV estimates from parameters derived from existing microstructural MRI techniques?

From diffusion MRI, there exist several models for estimating axonal density (e.g. CHARMED (Assaf and Basser, 2005) or NODDI (Zhang et al., 2012)) and axon diameter (AxCaliber (Assaf et al., 2008) and ActiveAx (Alexander et al., 2010)). Similarly, myelination can be estimated from relaxometry (Deoni et al., 2008) and magnetisation transfer imaging (Sled and Pike, 2001; West et al., 2016). Combining estimates of axonal and myelin volume fraction allows the generation of *in vivo* maps of g-ratio, for example, by combining, NODDI and qMT (Stikov et al., 2015), NODDI and mcDESPOT (Dean et al., 2016) or CHARMED and MTV (Duval et al., 2017). A recent review of MRI based g-ratio estimation is provided by Campbell et al. (2018).

All these techniques work by fitting microstructural parameters to biophysical models of the MRI signal using some numerical optimisation routine. This approach has some inherent issues. MRI signals are subject to noise from a range of sources. There are problems with fitting model parameters to MRI signals, including degeneracy of solutions in the optimisation process, and the likelihood of fitting the model to noise contributions. As a result, there can be considerable bias in MRI-derived microstructural metrics (Jones, 2010). We note, in particular, that quantification of inner AD is challenging, if not impossible, at gradient strengths found on typical clinical MRI system (up to 80 mT/m) (Burcaw et al., 2015; Lee et al., 2017; Nilsson et al., 2017; Veraart et al., 2019). This was a criticism levied at the study of Horowitz et al. (Innocenti et al., 2015; Lee et al., 2017). However, the advent of ultra strong gradient systems (300 mT/m) provides sensitivity to AD, at least over a limited but relevant range (i.e. above 3μm) (Nilsson et al., 2017). In this work we therefore focus on simulation (and real data) on an ultra strong gradient system. Although this is a special case, it does allow us to evaluate the feasibility of estimating CV *in vivo*.

This issue of model bias can become even more pernicious if some models take as input the output of other models, leading to propagation of noise and bias through different models. It is imperative, therefore, that MRI-derived estimates of CV are robust to such errors, which is the subject of investigation in the present study.

### 3.1 Method

To model the effects of MRI noise, MRI data were simulated using analytical expressions for three biophysical models, the Composite Hindered and Restricted Model of Diffusion (CHARMED) (Assaf and Basser, 2005), the AxCaliber model (Assaf et al., 2008) and multicomponent driven equilibrium single pulse observation of T1/T2 (mcDESPOT) relaxometry (Deoni et al., 2008).

### 3.2 Core biophysical simulations

A single population of axons whos AD distribution is parametrised by a continuous Poisson distribution (mean and s.d. of ADs is parameterised by *λ*) was simulated. The simulations assume no orientational dispersion or crossing fibre configuration. The biophysical parameters of the system are listed in Table 4. Systems with this configuration were simulated for a range of AVFs, axon diameters and g-ratios. The g-ratio value is treated as an aggregate estimate of g-ratio across the volume. 6 AVF values were tested from 0.1 to 0.7; 12 approximately evenly-spaced mean AD values from 0.5 to 12.5 μm and 11 evenly-spaced g-ratio values of 0.4–0.9.

**Table 4:** Fixed biophysical parameters used for the MRI simulations.

Fixed biophysical parameters		
Parameter	Units	Value
Intracellular axial diffusivity	${\text{cm}}^{2}\;{\text{s}}^{-1}$	2.0
Extracellular axial diffusivity	${\text{cm}}^{2}\;{\text{s}}^{-1}$	1.3
Extracellular radial diffusivity	${\text{cm}}^{2}\;{\text{s}}^{-1}$	0.15
Orientation (*θ*)	rad	π/2
Orientation (*ϕ*)	rad	0
Myelin $T_{1}$	ms	0465
Myelin $T_{2}$	ms	26
Myelin residence time	ms	180
Extracellular $T_{1}$	ms	1070
Extracellular $T_{2}$	ms	50
CSF $T_{1}$	ms	4000
CSF $T_{2}$	ms	2500
CSF volume fraction		0.05

#### 3.2.1 Diffusion MRI simulation

Diffusion MRI data were simulated using the AxCaliber model, utilising a population of van Geldren cylinder models (Van Gelderen et al., 1994) with a continuous Poisson distribution of ADs. The extracellular space was modelled as a zeppelin-shaped (cylindrically-symmetric) tensor. CHARMED and AxCaliber MRI data were simulated using the Microstructural Diffusion Toolbox (MDT) (Harms et al., 2017) using parameters that matched a standard protocol used on a Siemens 300 mT/s Connectom system (Jones et al., 2018) where a range of b-values and diffusion times were sampled (Table 3). The CHARMED and AxCaliber model were then fitted to the simulated data using the Powell optimisation routine (Powell, 1964) using the MDT toolbox.

#### 3.2.2 Relaxometry MRI simulation

mcDESPOT MRI data were simulated using the Quantitative Imaging Toolbox (QUIT) (Wood, 2018). The protocol comprised 8 spoiled gradient recalled (SPGR) images with varying flip angles and 16 steady-state free precession (SSFP) (Table 3) images distributed across 8 flip angles and 4 phase cycle angles. To account for the influence of radio frequency field strength ($B_{1}$) and off-resonance frequency ($F_{0}$) in the fitting, a range of $B_{1}$ and $F_{0}$ values were simulated for each noise measurement. To replicate the noise profile obtained from SNR measurements across flip angles, noiseless data were simulated and Rician noise with flip-angle-specific s.d. was added to the simulated data (see Appendix C). A 3-pool model (modelling contributions from myelin, extra-cellular and CSF water) was then fitted to the simulated data using the ‘qimcdespot’ function in the QUIT toolbox.

Since mcDESPOT gives a myelin water fraction (MWF) map, as opposed to a true MVF, we estimated the true MVF from the formula:

(7) $$\text{MVF}=\frac{\text{MWF}\left(1+\omega \right)}{1+\omega \text{MWF}}$$

where ω=0.72 is the ratio of lipid to water in the myelin (Agrawal et al., 2009) (see Appendix D for derivation). Similarly the axonal water fraction (AWF) obtained from the CHARMED model is not a true AVF. This can be obtained from:

(8) AVF=(1−MVF)×AWF

#### 3.2.3 g-ratio and CV estimation

g-ratios were computed using the approach of Stikov et al. (2015):

(9) $$g=\frac{1}{\sqrt{1+\frac{\text{MVF}}{\text{AVF}}}}$$

This approach has been shown to give a valid aggregate measure of g-ratio for a distribution of ADs. Using the Rushton model (Eq. (4)), with p=16.99 as fitted previously using simulations, CV was estimated for each combination of mean AD and g-ratio.

#### 3.2.4 Noise

Noise was simulated by adding Rician noise to each simulated MRI acquisition. The s.d. for each acquisition was modified to replicate the SNR profiles observed in real data (see Appendix C). Additionally, to test sensitivity to noise, data simulations were repeated with noise s.d. at 50% and 200% of the original noise s.d. This was done for all permutations across the 3 MRI parameters. For all simulated acquisition and permutations of noise levels, 100 iterations were performed. This resulted in a total of 100×3×6×10×11=198,000 diffusion MRI simulations and 100×3×6×11=19,800 relaxometry MRI simulations.

#### 3.2.5 Error measurements

Errors in CV estimates were quantified in the following ways:

1. Bias was quantified by the mean relative error in CV. The same was also done for the derived biophysical parameters required to compute CV (AVF, MVF, g and AD).
2. Variance was quantified by the variance of the CV estimates normalised to the original CV estimate.
3. Relative sensitivity to errors in parameter estimates was derived analytically, since the expression for CV is agnostic to the method used to estimate MWF and AWF and is easy to differentiate with respect to the initial fitted parameters (AD, MWF and AWF) by substituting equation (8) 7 9 into equation (4). The calculation of relative sensitivity is adapted from eq. (3) for analytical derivatives:

(10) S(ϕi)=∂v∂ϕi(Φ')|ϕ'iv(Φ')|

4. Sensitivity to noise was estimated using eq. (3) by taking the difference in CV estimates from simulations performed with 50% and 200% of the original noise level and normalising to the difference in noise s.d.

### 3.3 Results

#### 3.3.1 Errors in modelled parameters

**Fig. 6:**
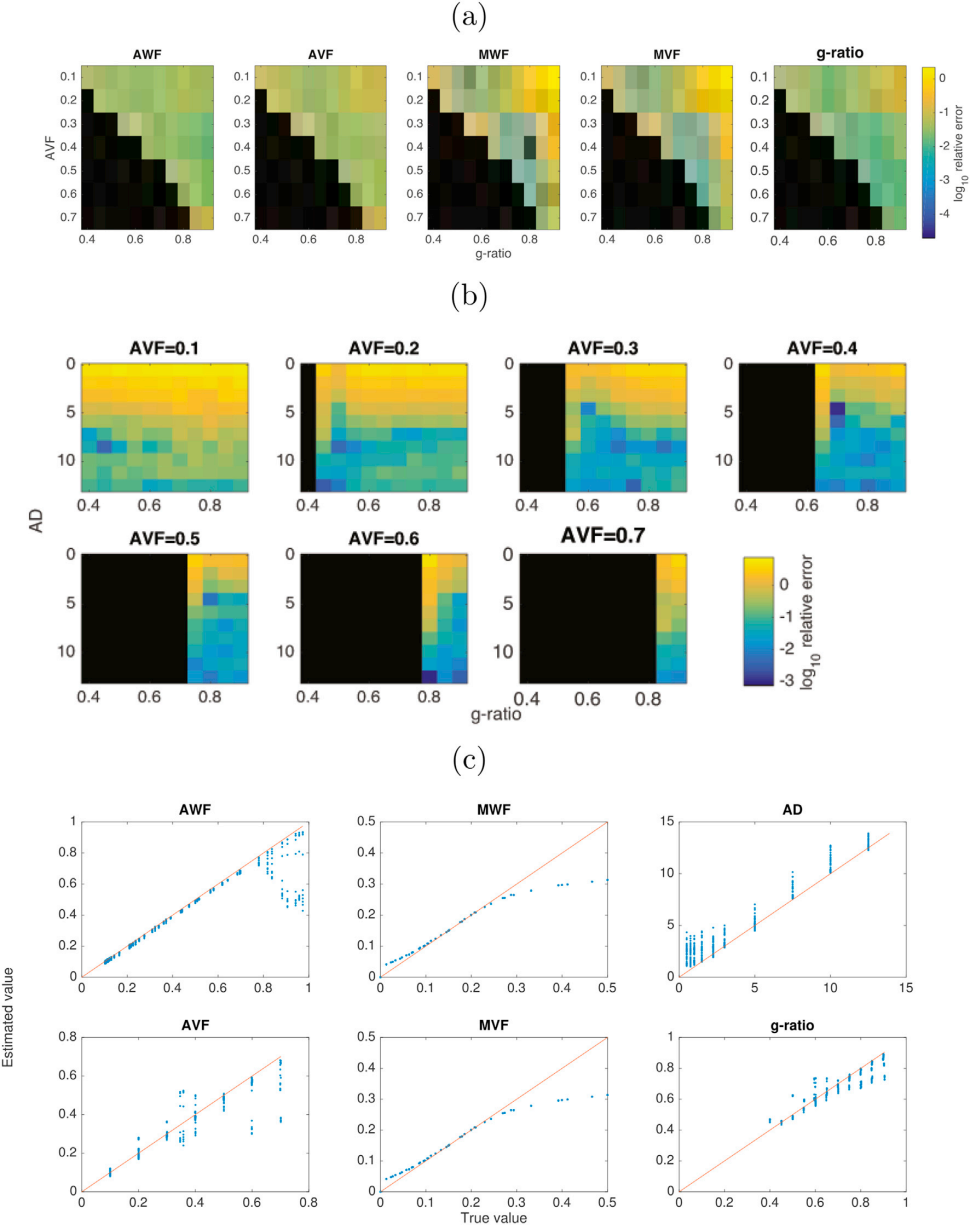
(a) Log relative errors in AWF, AVF, MWF, MVF and g-ratio across ranges of g-ratio and AVF. (b) log relative errors in AD, across ranges of AD, g-ratio and AVF. (c) true vs estimated values for all estimated variables.

**Fig. 7:**
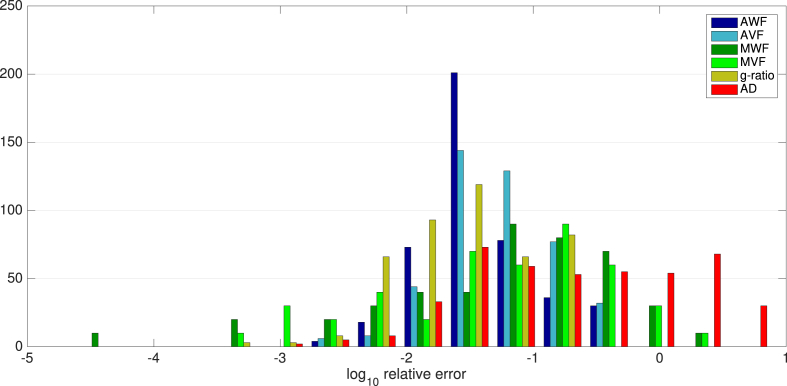
Distributions of log relative errors in derived parameters AWF (dark blue), AVF (light blue), MWF (dark green), MVF (light green), g-ratio (orange) and AD (red) across all parameters.

Errors in relevant fitted parameters are shown in Fig. 6 and distributions of errors across parameters are shown in Fig. 7. Overall, of the initially derived variables, the lowest errors are in the derived AWF (mean ± s.e.: 0.073±0.0003) with highest errors for AD (0.84±0.0029). MWF errors are 0.21±0.0009. Interestingly, errors in the derived g-ratios are lower than for its dependencies (0.054±0.0001). AWF and AVF have highest errors where g-ratio approaches infeasible values. AD values show the greatest errors where AD is low and AVF is low (i.e. where there is small contribution from intracellular diffusivity). There is a consistent positive bias in AD estimates, of up to 4 μm across all values of AD tested. Errors in g-ratio estimates are mostly uniformly low, but slightly higher errors where g-ratio is high and AVF is low. This corresponds to higher errors in the MWF/MVF estimates for these parameter values (where signal contributions from myelin water will be very small). g-ratio estimates are shown to have an increasing negative bias as g-ratio increases.

#### 3.3.2 Errors in CV estimates

**Fig. 8:**
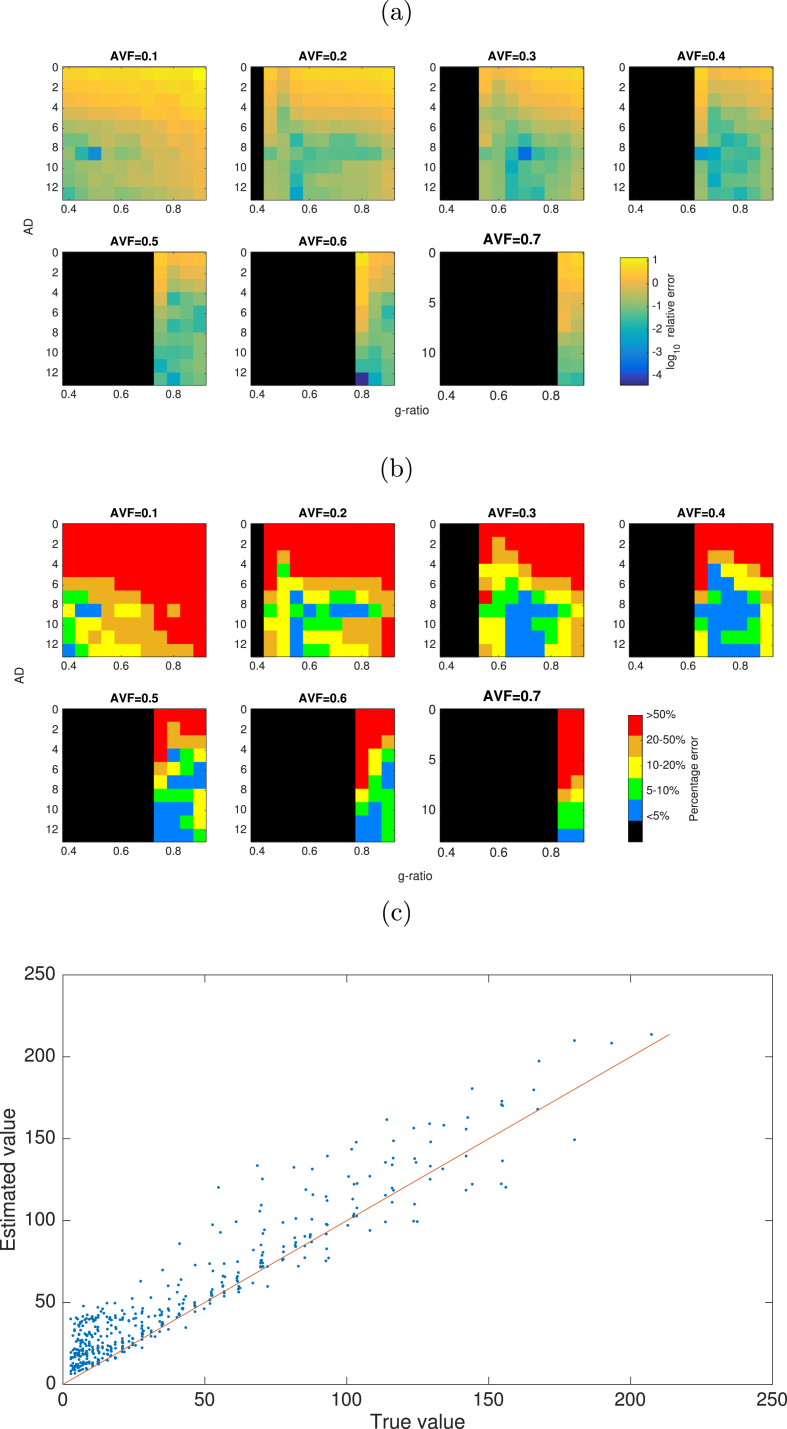
(a) Log relative error in CV estimates across values of AVF, AD and g-ratio. (b) Regions of parameter space where relative variance is less than 5% (blue), 5–10% (green), 10–20% (yellow), 20–50% (orange) and greater than 50% (red) error in CV estimates. Black regions are where axon AVF/g-ratio combinations gives an infeasible MVF values.

Relative errors in CV across the parameter space tested are shown in Fig. 8. The CV estimates show a less than 5% bias across a region of parameter space where AVF is 0.3 or above and AD is above 4μm. This boundary decreases slightly for AVF values of 0.4–0.6. There is little dependency on g-ratio except for very low AVF. Bias is greatest (over 50%) in regions where AVF is low (below 0.3) and AD is below 4μm. Examining the true vs estimated values show that there is generally a positive bias to CV estimates, of up to 50 ${\text{ms}}^{-1}$, with some negative estimates where true CV is between 0 and 150 ${\text{ms}}^{-1}$.

#### 3.3.3 Variance in CV estimates

**Fig. 9:**
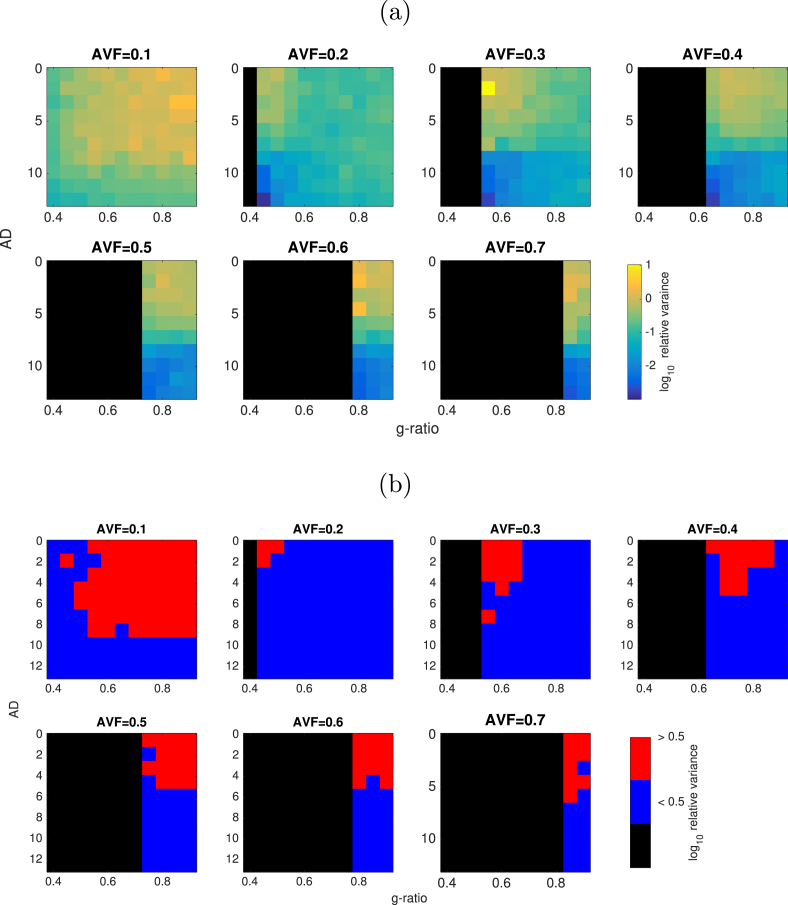
(a) Log normalised variance in CV estimates across values of AVF, AD and g-ratio. (b) Regions of parameter space where normalised variance is less than 0.5 (coloured in blue) or greater than 0.5 (coloured in red).

Variance in CV estimates is shown in Fig. 9. The normalised variance is mostly below 0.5 where AVF is above 0.1 and AD is higher than 4 μm.

#### 3.3.4 Sensitivity to parameter errors

The relative sensitivity to AD is easy to derive, since the Rushton expression is a linear function of AD, so relative sensitivity is 1 across the whole parameter space.

(11) S(d)=1

The relative sensitivities to AWF and MWF are more complex as CV are is not a simple linear function of AWF or MWF. Both sensitivities have dependencies on MWF and AWF, but not AD.

(12) $$S\left(\text{AWF}\right)=-\frac{\text{MVF}}{2\text{AVF}\left(\frac{\text{MVF}}{\text{AVF}}+1\right)\text{log}\left(\frac{\text{MVF}}{\text{AVF}}+1\right)}$$

(13) $$S\left(\text{MWF}\right)=\frac{\text{MVF}\left(\frac{\text{MVF}-\omega {\text{MVF}}^{2}}{\text{AVF}}-\frac{\omega \text{MWF}}{\omega \text{MWF}+1}+1\right)}{2{\text{AVF}}^{2}\left(\frac{\text{MVF}}{\text{AVF}}+1\right)\text{log}\left(\frac{\text{MVF}}{\text{AVF}}+1\right)}$$

**Fig. 10:**
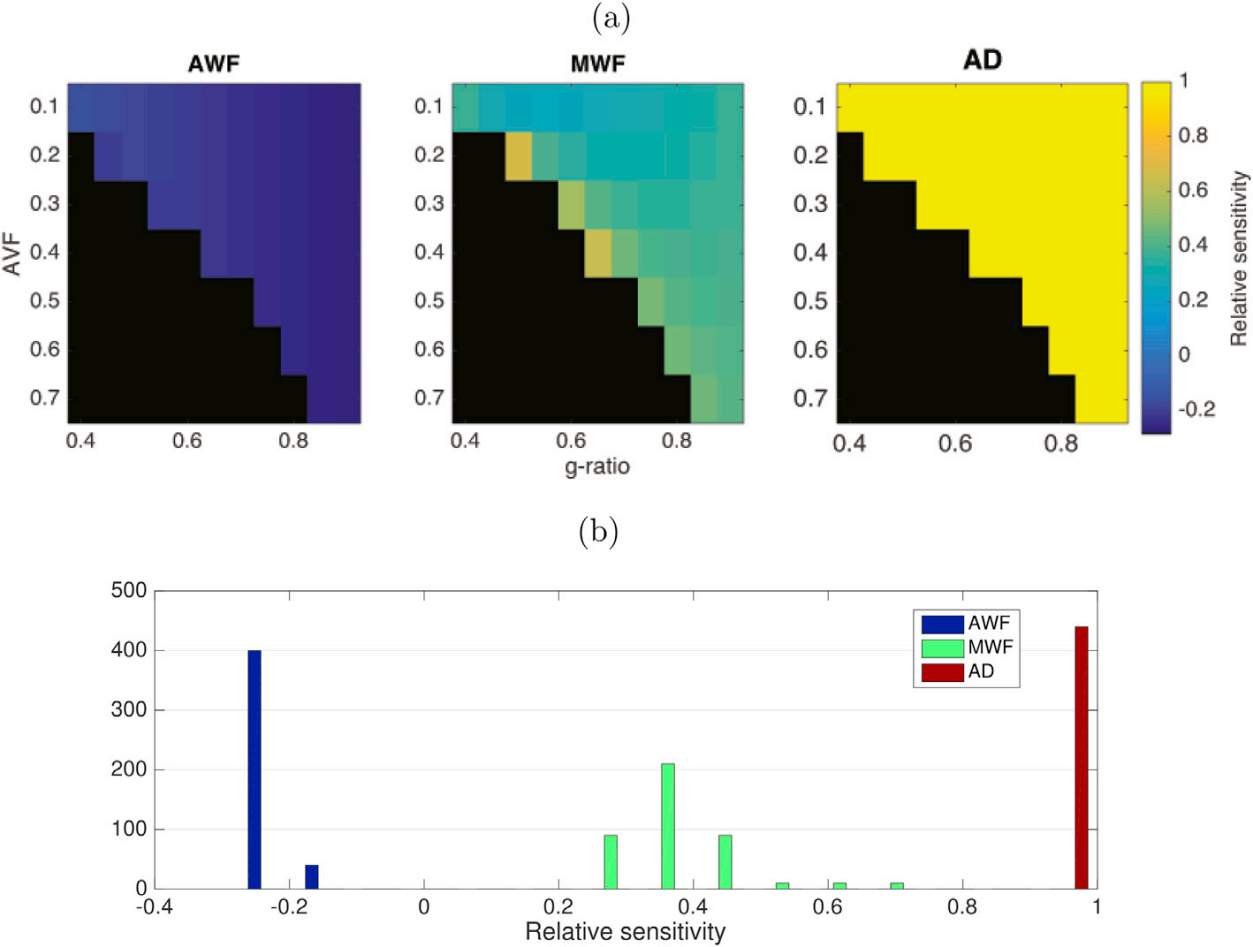
(a) Relative sensitivity (derived analytically) of CV estimate to errors in AWF, MWF and AD. (b) Distribution of relative sensitivities across the parameter space tested.

The relative sensitivities evaluated for the AVF and g-ratio values used in simulations are shown in Fig. 10. Relative sensitivity to AD errors is the highest with a uniform value of 1. Errors in AWF have the smallest effect giving a small negative bias in CV (−0.14±0.030). Errors in MWF give a moderate positive bias (0.38±0.088).

#### 3.3.5 Sensitivity to noise

**Fig. 11:**
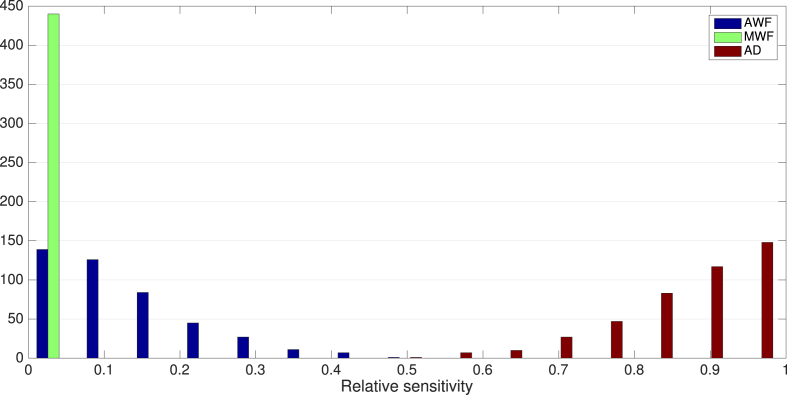
Distributions of log relative sensitivity of CV to noise in AWF (blue), AD (red) and MWF (green) acquisitions across all parameters.

**Fig. 12:**
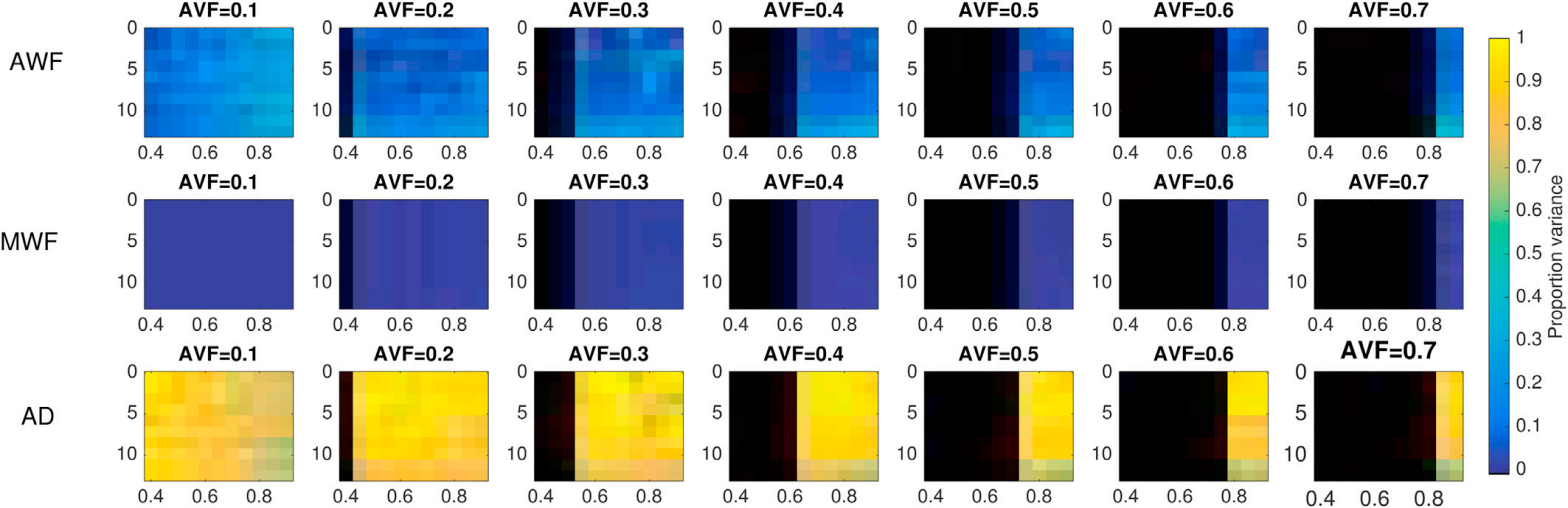
Proportional variance explained by noise in MRI acquisition, across the parameter space tested.

Distributions of relative sensitivities to noise across the tested parameter space are shown in Fig. 11. Proportional variances are quite uniform across the parameter space. The proportional variance in CV estimates explained by noise across three MRI parameters are shown in Fig. 12, which is also mostly uniform across the parameter space tested. CV has the highest relative sensitivity to noise in AD estimates (0.87±0.097). CV has the lowest relative sensitivity to MWF estimates (0.0031±0.0043). Relative senstivity of CV to noise in AWF estimates was also low (0.13±0.095).

### 3.4 *In vivo* CV estimates from human MRI data

As a proof of principle we apply the proposed approach to *in vivo* human data, subject to the caveats regarding the sensitivity to AD. Data were acquired on a high gradient MRI system. The analysis focuses on the corpus callosum as the axons here have a relatively uniform orientation and minimal dispersion.

### 3.5 Method

#### 3.5.1 MRI acquisition

CHARMED, AxCaliber and mcDESPOT data were all acquired from 21 healthy human participants (2M, 19F; 25.7±9.9 years of age) on a Siemens 3T 300 mT/m Connectom system (Siemens Healthcare, Erlangen, Germany). The acquisition parameters used were identical to those used in the simulations (see Table 3).

#### 3.5.2 Diffusion MRI processing

Motion, eddy current and EPI distortions were corrected using FSL TOPUP and EDDY tools (Andersson and Sotiropoulos, 2016). Correction for gradient non-linearities, (Glasser et al., 2013; Rudrapatna et al., 2018), signal drift (Vos et al., 2017) and Gibbs ringing artefacts (Kellner et al., 2016) was also performed. All diffusion data were then registered to a skull-stripped (Smith, 2002) structural T1-weighted image using EPIREG (Andersson and Sotiropoulos, 2016). AVF and AD parameters were fitted to the CHARMED and AxCaliber models using the MDT toolbox (Harms et al., 2017) using the same optimisation routine used in the simulations.

#### 3.5.3 Relaxometry MRI processing

Motion correction was applied to the SPGR and SSFP data using FSL mcFLIRT and then the brain was skull-stripped (Smith, 2002). All subsequent fitting steps were performed using the QUIT toolbox (Wood, 2018). A $B_{1}$ map was estimated by fitting the data to the DESPOT1-HIFI model (Deoni, 2007) and then fitting to a 8th order 3D polynomial. An $F_{0}$ map was estimated by fitting to the DESPOT2-FM model (Deoni, 2009). These were then used for the final fitting to the mcDESPOT model, as described for the MRI simulations. The final MVF maps were registered to the T1-weighted image using FLIRT (Andersson and Sotiropoulos, 2016) so that all parameter maps were in the same image space.

#### 3.5.4 Corpus callosum ROI

The corpus callosum was automatically segmented from the mid-sagital slice and divided into splenium, body and genu segments. The corpus callosum mask was eroded slightly with a disk kernel of radius 1.5 mm to minimise contributions from partial volume effects on the edge of the corpus callosum.

#### 3.5.5 CV mapping

AVF, MVF, g-ratio and CV parameters were computed from the modelled AWF, MWF and AD parameters, in the same way used in the simulations. In an attempt to overcome the bias of AD estimation, we used the simulation results to generate a spline-interpolated mapping between the biased and unbiased AD estimates, and used this to make a bias-corrected AD map and a subsequent bias-corrected CV map.

### 3.6 Results

**Fig. 13:**
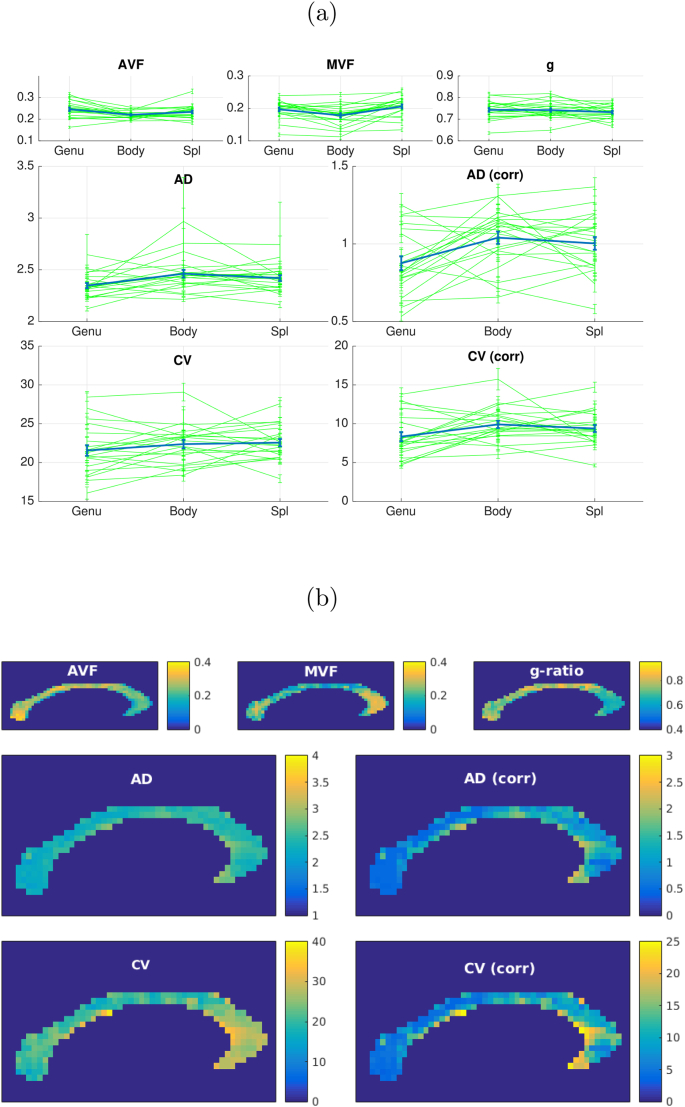
(a) Fitted *in vivo* MRI data (n = 21) to microstructural parameters in segments of the corpus callosum. Error bars show mean and s.e. in three main segments of the corpus callosum. Green show data for individual subjects, blue shows the group averaged data. (b) Fitted parameters in the corpus callosum in an individual subject.

*In vivo* MRI data in the corpus callosum are shown in Fig. 13. CV mean ± s.e estimates across all subjects are $21.6\pm 3.1\;\text{m}\;{\text{s}}^{-1}$ in the genu, $22.4\pm 2.5\;\text{m}\;{\text{s}}^{-1}$ in the body and $22.6\pm 2.8\;\text{m}\;{\text{s}}^{-1}$ in the splenium. The bias corrected CV estimates are $8.3\pm 2.7\;\text{m}\;{\text{s}}^{-1}$ in the splenium, $9.9\pm 2.2\;\text{m}\;{\text{s}}^{-1}$ in the body and $9.4\pm 2.1\;\text{m}\;{\text{s}}^{-1}$ in the splenium.

There is a distinct profile of highest CV estimates in the body compared to the genu, which is consistent across most subjects. There are slightly higher mean values in the splenium compared to the genu. The bias-corrected CV estimates are overall lower than the uncorrected estimates, but still are slightly high compared those measured in electrophysiology in primates (median value of $7.4\;\text{m}\;{\text{s}}^{-1}$) (Swadlow et al., 1978) or estimated from primate histology ($5.4-8.9\;\text{m}\;{\text{s}}^{-1}$) (Caminiti et al., 2013).

## 4 Discussion

This work has explored the feasibility of obtaining conduction velocity (CV) maps from *in vivo* human MRI, using a simplified model of axonal CV. Results from the axon simulations demonstrate that 85.1% of the variance in CV, and 94.6% of the sum-squared sensitivity of CV, can be attributed to variance in AD and g-ratio. Examining the proportional variances (using ecologically valid variances and covariances in parameters where possible), implicate AD as the most important parameter, while looking at sensitivity to a unit change in parameter, g-ratio is implicated as the most important. Therefore, considering the fact that AD varies much more in axon populations than g-ratio, capturing accurate estimates of AD is clearly more important than g-ratio for CV estimation.

The Rushton (1951) and outer diameter (Waxman and Bennett, 1972) models for CV both provide a reliable estimate of CV from MRI-derived estimates of g-ratio and AD. In addition, we show that it is possible to account for uncertainty in CV estimates due to parameters not accessible *in vivo*. Thus, when reliable estimates of AD and g-ratio can be made, it is feasible to obtain estimates of axonal CVs *in vivo*. The match in the parsimony measures (AIC/BIC) for the Rushton model and outer-diameter model (Table 2) were comparable, with a slight improvement in SSE for the Rushton model. Indeed, Caminiti et al. (2013) used the outer-diameter model to good effect (see also Lee et al. (2017) for a discussion of the merits of the outer diameter model). Examining the regional difference in the SSE (Fig. 5) it is shown that for g-ratios in the range 0.5–0.75 the Rushton model performs slightly better. Performance is better for the outer diameter model for large g-ratios. Given that most axons conform to the former range of g-ratio (Stikov et al., 2015), the Rushton model is the preferred approach, and thus an estimate of both inner diameter and g-ratio is valuable for mapping CV. It should be noted this primarily affects larger axons. With smaller axons, the two models are comparable in performance.

More complex models of CV derived using a polynomial expansion gave better fits than the two simpler models, but the parsimony measures indicate that, due to the increased number of coefficients, these models could be over-fitting the data. Fig. 4, shows that the main improvement in the polynomial models is in regions where AD is high and g-ratio is low. However, such axon configurations are uncommon: large diameter axons are unlikely to have very thick myelin sheaths. Therefore, in practice, there is little value gained by employing these more complex models to estimate CV *in vivo*.

In terms of sensitivity to errors in parameter estimation, we investigated the effects of bias in MRI-derived parameters, the sensitivity of CV estimates to these errors, and the sensitivity to noise. Overall we show that in regions where mean AD is high, the errors in CV estimates are typically below 10% over a large region of the parameter space. The threshold below which AD causes large errors in CV (above 50%) varies with AVF: about 5μm for AVF of 0.1, about 3μm for AVF of 0.3–0.6. For human CNS, this is problematic since ADs in human CNS are typically below 1 μm (Aboitiz et al., 1992; Liewald et al., 2014; Caminiti et al., 2013; Sepehrband et al., 2016a). This limits the potential relevancy of CV estimates to human *in vivo* data (see section below for further discussion). Interestingly, the range of g-ratios at which CV estimates are optimal shifts upward as AVF increases. The same effect can be seen for g-ratio errors in Fig. 6. CV estimates were least accurate when the AVF is very small (0.1 or below). This is to be expected as more sparse axon populations will generate less signal and reduce performance of model fitting. Sensitivity to errors in AVF and MWF are comparatively low. However, CV is most sensitive to errors in AD. This is as expected, since the Rushton model has the highest sensitivity to AD. This, emphasise further the challenge CV estimation faces from poor AD estimation. This sensitivity is uniform across the parameter space, indicating that accurate AD estimation is critical for CV estimates, regardless of specific axon configurations.

### 4.1 Challenges faced by AD estimation

The most problematic aspect of estimation of CV, highlighted here, is the estimation of AD from dMRI. Since AD accounts for the most variability in CV, this presents a challenge to estimating CV from *in vivo* MRI. This was a major issue with the study of Horowitz et al. (2015). In the study of Berman et al. (2019), the issue was circumvented by using ADs sampled from distributions derived from existing histology (Aboitiz et al., 1992). Given that our electrophysiological simulations show AD account for the most variance in CV, one would need to be able to capture individual-specific estimates of AD, to properly estimate individual-specific CV. Although they observed a small difference in CV between older and younger subjects, the authors conclude that *in vivo* AD estimates are necessary since this effects how much g-ratio contributed to CV. Using external population-level histological estimates of AD in the CV calculations, neglects any possible individual differences in AD. Methodological issues around the estimation of axon diameters must therefore be addressed to ensure accurate CV estimation across the whole CNS. Our attempts to estimate CV in corpus callosum, although showing a similar profile observed in the literature (Caminiti et al., 2013), still result in a small positive bias compared to literature values, even after attempts to correct this bias. AD estimates are higher than expected while g-ratio estimates are in the expected range, consistent with the findings from our simulations.

The apparent inter-axonal diffusion perpendicular to the axon (which is used to estimate AD) is orders of magnitude smaller than the apparent extra-axonal diffusion (Burcaw et al., 2015; Lee et al., 2017; Veraart et al., 2019). This is the main challenge to estimating smaller ADs, and requiring acquisitions at high *b* values to ensure a non-negligible contribution from the intra-axonal space (Veraart et al., 2019). On clinical MRI systems with gradients of up to 70 mT/m, this is problematic. On such systems, ADs below 6μm will not be detectable. However, on a high gradient system (300 mT/m), where high *b* values are achievable, this can be reduced to 2–3 μm (Drobnjak et al., 2016; Sepehrband et al., 2016b; Nilsson et al., 2017). This is still not good enough given that the majority of axons in the brain are lower than 1 μm (Aboitiz et al., 1992; Liewald et al., 2014; Caminiti et al., 2013; Sepehrband et al., 2016a) and so obtaining accurate estimates of axon diameter across the whole brain is not currently possible. In our in-vivo data we attempted to correct for this positive bias, by creating a simple mapping between the biased and unbiased ADs from our simulations. Although AD estimates were closer to expected values, they were still high. Furthermore, this mapping is ill-conditioned as any true sub-resolution ADs will be estimated at the limit (about 2.5μm in our *in vivo* data). Fortunately, some recent preliminary evidence suggests that, even if the majority of axons are below the resolution limit, there is still sufficient signal from the larger axons at the tail of the distribution to allow us to extrapolate the shape of the distribution below this limit, and hence derive a more accurate mean AD (Drakesmith et al., 2018; Chiappiniello et al., 2018; Dell’Acqua et al., 2019). If proven to be reliable, this would represent significant progress to improving AD, and hence CV estimation *in vivo* and warrants further exploration. Furthermore, development of MRI hardware with even stronger gradients of 500 mT/m (Basser et al., 2018) lends hope to the AD resolution limit can be pushed even further down. Nevertheless, we stress that currently measuring AD remains challenging and we are not suggesting that it is possible to estimate CV everywhere within the brain (Lee et al., 2017).

An alternative to estimating internal AD is the framework of Novikov et al. (2014, 2018); Fieremans et al. (2016); Burcaw et al. (2015); Lee et al. (2017) which allows characterisation of diffusion in the extra-axonal space in terms of the packing geometry of axons, which is dependent on the outer fibre diameter. This is appealing as this is closely correlated to CV, and only requires estimation of one microstructural parameter, instead of two, as used by the Rushton model. Lee et al. (2017) suggest that the apparent correlation between AD and CV observed by Horowitz et al. (2015) is due to contributions from the extra-axonal diffusion to the signal not being modelled correctly. However, it is still unclear how outer fibre diameter can be disentangled from other geometric properties of the extra-cellular space (e.g. packing density and packing randomness) within this framework. Also, as highlighted in the present study, the Rushton model is more accurate than the outer diameter model for estimating CV over a more common range of g-ratios. However, the merits of this modelling framework deserve to be explored further.

### 4.2 Other considerations for MRI methods

The estimated CV is assumed to be a valid aggregate measure of CV for a population of axons. The mean AD is parameterised by *λ* of the Poisson distribution and the g-ratio calculation has been shown to be valid for a distribution of ADs (West et al., 2016). However, it is unclear if the CV value obtained from aggregated AD and g-ratio values is a valid aggregate representation of a distribution of CVs. What represents the most optimal parameterisation of the AD distribution also remains an open question. A continuous Poisson distribution was chosen in this work as it has only one parameter that characterises both the mean and standard deviation, thereby reducing model complexity and improve optimisation. However, other distributions may offer better approximations of distributions observed in histology (Sepehrband et al., 2016a).

An additional challenge that remains unexplored is the estimation of AD in other white matter pathways, where there are multiple regions of fibre crossing and dispersion. In the present study, these configurations were not considered. However, such configurations can be challenging for models that assume a single fibre geometry, such as AxCaliber. It is possible to model multiple fibre populations (Barazany et al., 2011), but this substantially increases the number of parameters to estimate. The issue of dispersion can potentially be resolved by including a dispersion term into the AxCaliber model as done in the NODDI model. The Convex optimisation modelling for microstructure informed tractography (COMMIT) framework (Daducci et al., 2015) can estimate microstructural properties along a tractogrpahy streamline, assuming the parameter does not vary along the length of the tract. This will allow estimation of distinct axon diameters for distinct fibre populations.

We also note that different combinations of methods can yield different levels of accuracy in CV estimates. There numerous combinations of methods for estimating AWF and MWF that could be used to generate g-ratio maps, all with different advantages and limitations (Ellerbrock and Mohammadi, 2018; Campbell et al., 2018). In addition, different methods for estimating AD such as ActiveAx (Alexander et al., 2010) and time-dependent AxCaliber (De Santis et al., 2016) expands the number of permutations of methods even further. Further work is necessary to assess which combination produces the best estimates, similar to that of Ellerbrock and Mohammadi (2018). Previous work has shown there is an inherent bias in mcDESPOT (Lankford and Does, 2013; West et al., 2019). Our simulations also show (Fig. 8(c)) a negative bias in MWF estimates for higher MWF values. However, our sensitivity analysis also shows that CV estimates have low sensitivity to MWF errors. While this study explored the impact of MRI noise on CV estimates, there is a number of other sources of confounding variance, such as motion, eddy currents, field inhomogeneities and gradient non-linearities that could impact on CV estimates to differing degrees.

### 4.3 Considerations for electrophysiological simulations

While efforts have been made to incorporate true biological variability in the sensitivity analysis by taking parameter ranges from the literature, where available, the simulations are currently restricted to a single axon population. Variability should be considered across axon populations, where, for example, it is known that AD varies considerably throughout the CNS (Perge et al., 2012) as well as along single axons (Tomassy et al., 2014). Similarly within-axon variation in g-ratio, internodal length and nodal diameter also exist (Ford et al., 2015). However, it is important to keep in mind that at the spatial resolution of MRI, (e.g. $2\times 2\times 2{\text{mm}}^{3}$), we effectively average the axonal properties over thousands of axons and, as such, such variations will be averaged out to a certain extent. Cross-correlations between axon parameters should also be considered. In our simulations, we forced such correlations between internode and nodal AD, and between AD and internodal length, since such relationships are well documented in the literature (Waxman, 1980). However, other cross-correlations are likely to exist in nature but not simulated here.

Despite these possibilities for future improvements, our simulations produced a constant of proportionality between fibre diameter and CV of 6.67 $\text{m}\;{\text{s}}^{-1}/\text{μm}$, which is only slightly above the range of 5.5–6.0 $\text{m}\;{\text{s}}^{-1}/\text{μm}$ commonly reported in the literature (Gasser and Grundfest, 1939; Hursh, 1939; Rushton, 1951; Smith and Koles, 1970; Waxman and Bennett, 1972; Tolhurst and Lewis, 1992). Looking further at Fig. 4(d), it looks like fitting only axons with diameters less than 5μm would result in a smaller constant of proportionality. We are therefore confident we have captured the inter-relationships between parameters reasonably well. We also note that the strong contribution of INL to CV would mean neglecting the AD-INL correlation would result in a significant underestimation of this constant.

A key assumption made here is that the results of the axon simulations, whose baseline parameters are based on rat optic nerve (Arancibia-Cárcamo et al., 2017), are generalisable to other white matter axons, and to other species. an entirely valid assumption. For example, membrane capacitance has recently been shown to be lower in human neurons compared to rat neurons (Eyal et al., 2016). Moreover, there is a theoretical optimal g-ratio for a given fibre diameter (Smith and Koles, 1970). In the present sensitivity analysis, the range of g-ratios tested is in an interval where the relationship between CV and g-ratio is monotonic and approximately linear. However, for other fibre populations with different ranges of g-ratios where the relationship is non-monotonic, the sensitivities may differ substantially. A potential future research avenue is to repeat the sensitivity analysis on a range of axon populations to see which populations better lend themselves to modelling with MRI. However, obtaining all the morphological and electrophysiological parameters for multiple populations presents significant practical challenges.

It has been demonstrated that the relative thickness of the water and lipid layers in myelin vary with age (Agrawal et al., 2009), and consequently the assumed constancy of *ω* used in Eq. (7) may not be valid. This issue can potentially be resolved by combining multiple myelin-sensitive contrasts, e.g. by adding in quantitative magnetisation transfer (qMT) (Sled and Pike, 2001). While qMT does not provide unique sensitivity to lipids, it does have sensitivity to protons bound to lipids and macromolecules. It may therefore be possible to exploit qMT and relaxometry methods together to better characterise the water-lipid ratio in myelin.

### 4.4 Conclusion

We have demonstrated the feasibility of estimating CV for ensembles of axons from their diameter and g-ratio, estimated from *in vivo* microstructural MRI, provided axon diameters are sufficiently large to be modelled accurately. Difficulties associated with estimating smaller axon diameters present the largest challenge to CV estimation across the whole CNS. However, potential solutions are in development which will greatly improve the accuracy of MRI-based CV estimation.

## Figures and Tables

**Table A.1:** Changes in CV due to changes of parameters previously reported in (Arancibia-Cárcamo et al., 2017).

Parameter changed	Value	Relative change from baseline	CV ( $\text{m}\;{\text{s}}^{-1}$)	Relative change from baseline
Node length (nm)	0.5	−0.51	2.53	−0.14
	2.2	1.16	3.02	0.02
Fast Na ​+ ​conductance ($\text{mS}{\text{cm}}^{-2}$)	21	−0.30	2.64	−0.11
Number of wraps^†^	6	−0.14	2.66	−0.10

^†^, Number of wraps, AD and g-ratio were fixed for this simulation. This is equivalent to setting myelin periodicity to 18.5 nm (a relative change from baseline of 0.19).

**Figure B.1:**
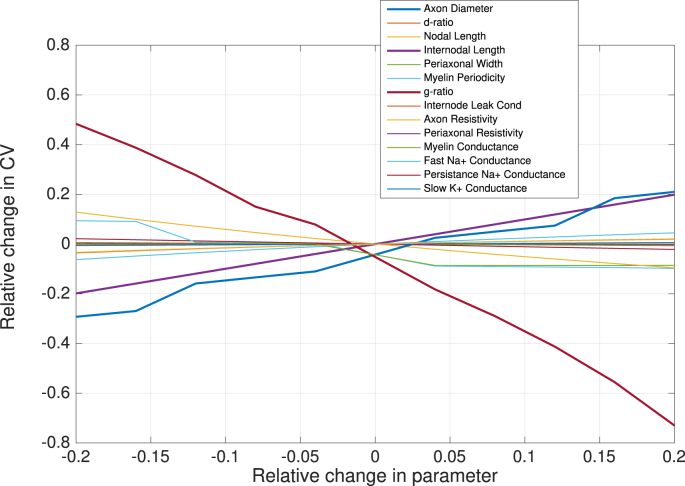
Results of OAAT analysis of sensitivity of CV to each of the free parameters tested.

**Figure B.2:**
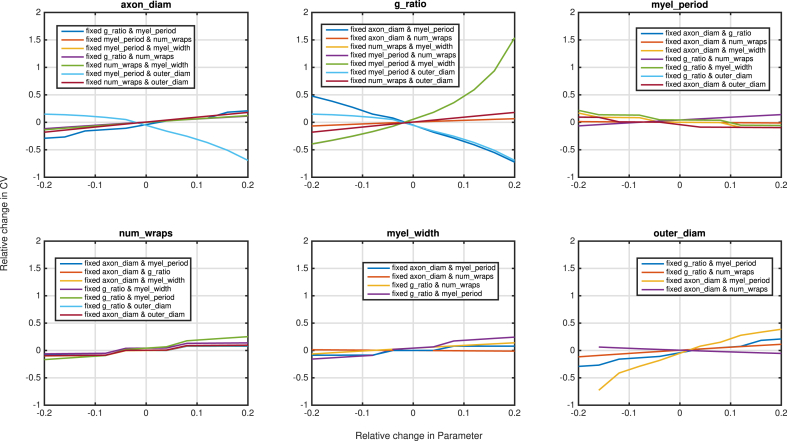
Results of OAAT analysis for the 6 interdependent geometric parameters with different combinations of parameter fixings.2

**Table C.1:** *In vivo* measurements of SNR and corresponding s.d. values for acquisition sequences used for estimating CV. S.D. values quoted for mcDESPOT scans are the effective noise added to recreate SNR measured *in vivo*.

Sequence	Flip angle (^o^)	SNR	noise s.d.
CHARMED	90	2690	0.019
AxCaliber	90	1776	0.024
mcDESPOT - SPGR			
	3	2246	0.134
	4.5	2701	0.087
	6	2805	0.069
	7.5	2670	0.064
	9	2394	0.062
	12	1873	0.056
	15	1044	0.068
	18	495	0.098
mcDESPOT - SSFP			
	10	10,136	0.010
	15	14,256	0.008
	20	17,666	0.008
	25	19,090	0.007
	30	19,659	0.007
	40	18,269	0.007
	50	14,272	0.008
	60	9776	0.010

**Figure D.1:**
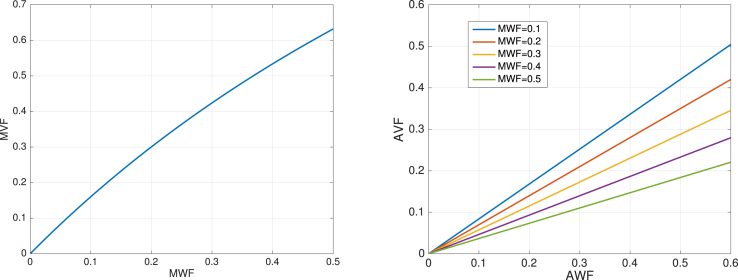
Theoretical relationships between MWF and MVF according to Eq. (7) (left). and between AWF and AVF according to Eq. (8) (right).3

## Appendix A Validation of axon model

To ensure the implementation of the “Model C” axon model (Richardson et al., 2000) produces results consistent with Arancibia-Cárcamo et al. (2017), simulations were carried out with the baseline condition and some parameters varied as described in this paper. All other model parameters were the same as used in the main simulations except that the stimulus current was fixed at 3 nA, as done in Arancibia-Cárcamo et al. (2017). The baseline condition produced a CV of 2.95 $\text{m}\;{\text{s}}^{-1}$, consistent with the results of Arancibia-Cárcamo et al. (2017). The results for other parameter variations are shown in Table A1 which are also consistent with those reported in Arancibia-Cárcamo et al. (2017).

## Appendix B OAAT sensitivity analyses

A one-at-a-time (OAAT) sensitivity analysis was performed for each parameter at 10 equally-spaced intervals within a ±20% range around the baseline condition. Results are shown in Figure B1, which shows all sensitivity to all parameters is approximately linear over the interval tested. In the main analysis, three of a set of six interdependent geometric parameters were manipulated: axon diameter, g-ratio and myelin periodicity. Three other parameters depend directly on these parameters: number of myelin wraps, myelin width and outer fibre diameter. As the impact of variance in these parameters will vary depending on which combinations of these parameters are fixed, we repeated the OAAT analyses for different combinations of fixings. The results are shown in Figure B2. For most combinations, the sensitivity to each parameter shows a trend in the same direction regardless of which combination of other parameters are fixed. Notable exceptions are for the g-ratio where the direction of the sensitivity varies depending on which parameters are fixed. Sensitivity to g-ratio shows a negative trend when axon diameter and myelin periodicity or axon diameter and number of wraps are fixed. Sensitivity to g-ratio shows a positive non-linear trend when myelin periodicity and myelin width are fixed.

## Appendix C SNR measurements

SNR for the diffusion sequences used for the AxCaliber and CHARMED acquisitions and the SPGR and SSFP acquisition used for mcDESPOT were measured by acquiring two image volumes: (1) a standard image volume (for diffusion sequences, only a $b=0\;\text{s}{\text{mm}}^{-2}$ image was used as the noise distribution is not expected to be affected by the level of diffusion weighting) with the standard flip angle (see Table 3); and (2) a noise image volume acquired with exactly the same parameters but with the flip angle set to 0, such that there is effectively no echo received by the receiver coil. The noise s.d. was computed from the signal magnitude of the noise volume. The results are shown in Table C1.

## Appendix D Estimating volume fractions from water fractions

mcDESPOT gives a myelin volume fraction (MWF) that is the proportion of the total water volume that resides in the myelin layers.

(D.1) $$\text{MWF}=\frac{\text{MWV}}{W}$$

whereas calculation of g-ratios requires the myelin volume fraction (MVF), which is the proportion of the total volume that is myelin (both water and lipid components):

(D.2) $$\text{MVF}=\frac{\text{MWV}+\text{MLV}}{V}$$

where MWV is the myelin water volume, MLV is the myelin lipid volume, *W* is the total volume of water and *V* is the total volume. If we assume that the ratio of water and lipid in the myelin compartment, *ω*, is constant, we can also express the MLV as:

(D.3) MLV=ωMWV

the total volume is given by:

(D.4) V=MWV+MLV+AWV+EWV

where AWV and EWV are the volumes of axonal and extracellular water, respectively. The total water is given by:

(D.5) W=MWV+AWV+EWV

Assuming the only non-water compartment is the myelin phospho-lipid layers and that the contribution of phospho-lipid membranes from other cell types is negligible, this can be expressed as:

(D.6) W=V−MLV

Substituting this into the expression for the MWF gives:

(D.7) $$\text{MWF}=\frac{\text{MWV}}{V-\text{MLV}}=\frac{\text{MWV}}{V-\omega \text{MWV}}$$

Collecting terms of MWV and rearranging gives:

(D.8) $$\text{MWV}=\frac{V\text{MWF}}{1+\omega \text{MWF}}$$

We can get an equivalent expression for the MLV by multiplying through by *ω*

(D.9) $$\text{MLV}=\frac{V\text{MWF}}{\frac{1}{\omega }+\text{MWF}}$$

Substituting these into the expression for MVF gives:

(D.10) $$\text{MVF}=\frac{\text{MWF}}{1+\omega \text{MWF}}+\frac{\text{MWF}}{\frac{1}{\omega }+\text{MWF}}=\frac{\text{MWF}\left(1+\omega \right)}{1+\omega \text{MWF}}$$

The value of *ω* was taken from Agrawal et al. (2009) where the width of the lipid bilayers was about 4.6 nm and the intra- and extracellular water layers were each 3.2 nm, giving a lipid-water ratio of ω=4.6/(3.2+3.2)=0.72. The relationship between MWF and MVF is show in Figure D1. This approach is similar to that used by Jung et al. (2018) although *ω* is parameterised slightly differently. The relationship between MWF and MVF derived here is in close agreement with that study. One difference to note is we assume *ω* to have no dependence on the number of myelin wraps. We note, for cases where the inner diameter is orders of magnitude greater than the width of the laminae (e.g. 0.82μm vs 15.6 nm in our axon model) and there are sufficient number of wraps (above 5), which is the case for the vast majority of white-matter axons, this dependence can be assumed to be negligible.

The axonal volume fraction (AVF) can then be obtained from the axon water fraction (AWF) given by the CHARMED model (denoted here ${\text{AWF}}_{d}$). The AWF obtained from CHARMED excludes contributions from myelin water due to the short T2 of this compartment and excludes contribution from myelin lipid due to insensitivity to contributions from myelin macromolecules. We therefore used the estimated MVF computed previously to obtain the AVF.

(D.11) $${\text{AWF}}_{d}=\frac{\text{AWV}}{\text{AWV}+\text{EWV}}=\frac{\text{AWV}}{V-\text{MWV}-\text{MLV}}=\frac{\text{AVF}}{1-\text{MVF}}$$

The AVF can therefore be obtained from the MVF estimated from mcDESPOT and the AWF given by CHARMED:

(D.12) $$\text{AVF}=\left(1-\text{MVF}\right)\times {\text{AWF}}_{d}$$
